# Impact of a virtual educational intervention on knowledge and awareness of biomedical waste management among Peruvian dental professionals

**DOI:** 10.1038/s41598-023-49878-5

**Published:** 2023-12-15

**Authors:** César Cayo-Rojas, Gissela Briceño-Vergel, Nancy Córdova-Limaylla, José Huamani-Echaccaya, Manuel Castro-Mena, Paolo Lurita-Córdova, Judit Bermúdez-Mendoza, Clifford Allen-Revoredo, Jorge Torres-Vásquez, Marysela Ladera-Castañeda

**Affiliations:** 1 School of Stomatology, Universidad Privada San Juan Bautista, Lima, Peru; 2 School of Stomatology, Universidad Privada San Juan Bautista, Ica, Peru; 3 School of Communication Sciences, Universidad Privada San Juan Bautista, Lima, Peru

**Keywords:** Health care, Dentistry, Disease prevention, Public health

## Abstract

Waste from healthcare is a significant global issue, with around 85% of it being common waste and the remaining 15% being hazardous waste that is infectious and toxic. Dentistry uses various materials that create a substantial amount of biomedical waste capable of impacting the environment. Therefore, the purpose of this study was to assess the effects of a virtual educational program on the knowledge and awareness of dental material recycling and reuse, as well as biomedical waste management, among dental professionals in Peru. The current study was a longitudinal and quasi-experimental evaluation of 165 dentists from Peru. A validated questionnaire consisting of 30 items was administered at three different intervals (pre-test, immediate post-test, and 14-day post-test). Statistical analysis was conducted using the Mann Whitney U and Kruskal Wallis H tests to compare scores between categories of each sociodemographic variable, and the Cochrane’s Q and Friedman test was used for related measures comparison. A significance level of *p* < 0.05 was considered. When comparing the percentage of correct responses regarding recycling and reuse of dental materials and biomedical waste management between the pre-test and the immediate post-test, a significant improvement in knowledge was observed for most of the questionnaire items (*p* < 0.05). At 14 days after the test, those who studied at a private university, unmarried, bachelors, non-specialists, non-teachers and have less than 10 years of professional experience did not did not retain knowledge on biomedical waste management (*p* < 0.05) or recycling and reusing dental materials (*p* < 0.05) to a significant extent. There was a significant enhancement in dentists' knowledge and awareness of managing biomedical waste, recycling, and reusing dental materials following the educational intervention. This improvement was observed across all sociodemographic variables considered in the study. However, this knowledge was not retained beyond two weeks for those who studied at a private university, unmarried, bachelor, with no specialty, non-teachers and with less than 10 years of professional experience. Government authorities should encourage oral health professionals to conduct research with educational interventions focused on improving and evaluating the sustainability and environmental impact of dental practices. This will enable professionals to better understand, control and evaluate the consequences of their practical work.

Waste from healthcare is a global issue. Out of all healthcare waste, 85% is non-hazardous, while the remaining 15% is capable of being infectious and poisonous^1^. It is estimated that 16 billion injections are administered annually, but the disposal of all needles and syringes is not always appropriate^2,3^. The most frequent injuries in healthcare are due to sharps. Thus, students and professionals may be exposed to various infections including severe acute respiratory syndrome coronavirus 2 (SARS-CoV2), hepatitis B (HBV), hepatitis C (HCV), human immunodeficiency virus (HIV), tuberculosis, and tetanus^1,4–6^.

According to the World Health Organization (WHO), hazardous waste makes up approximately 15% of harmful substances present in human habitats and the natural environment^1^. Due to numerous factors, including the volume of blood, saliva, and tissue removed during surgery, the pathogenicity of infectious agents, and the clinical conditions of both the patient and operator, as well as the safety measures used before, during, and after treatment, it is crucial to follow the protocols set out for managing biological waste. It is important to note that all patients attending dental offices should be considered possible carriers of infectious diseases due to the significant number of pathogenic microorganisms that inhabit the oral cavity^7,8^. Despite this, there is still a significant lack of knowledge and awareness in this area, resulting in a high risk of accidental exposure to contaminated materials^9,10^. According to reports, these incidents are often associated with risky behaviors such as improper disposal of sharps or inadequate transport and handling by professionals, students, and staff responsible for managing these wastes^11–14^.

Dental schools, as professional training centers and providers of health services to the community, bear the responsibility to prevent diseases and minimize environmental risks associated with waste generation during clinical care^3,11,15^. Hence, it is crucial to equip upcoming professionals not just with the skills to diagnose and treat diseases, but also to raise awareness about various issues relating to public health and the planet^5^. Encouraging intervention programs is an effective approach to promoting awareness and understanding of recycling and reusing biomedical waste^11^. The process of "reuse" involves frequent direct utilization of renewable resources either in their original form or after restoration, whereas "recycling" requires alteration of their original form or combination with other materials^16^.

Currently, numerous countries have expressed concern about the rising prevalence of diseases and climate change across the globe. Consequently, they have suggested implementing biosafety programs within the healthcare sector^17–19^. Despite the potential risks associated with biocontaminated waste, limited research exists on its management and correlation to disease occurrence in exposed populations in developing countries such as Peru. Studies have shown that ineffective disposal of biological waste remains prevalent in healthcare facilities, posing a potential risk for disease transmission^20,21^.

At the conclusion of dental treatment, it is imperative that waste materials be identified, categorized, and eliminated in accordance with the regulations stipulated by each country's Ministry of Health. These guidelines are critical for dental students, practitioners, and waste management staff to prevent the spread of infections and pathogens found in waste materials^22,23^.

Asiri et al.^24^ conducted a study revealing that the level of knowledge on dental waste management among dental health professionals was influenced by gender, work setting, age, and experience. Similarly, Puri et al.^25^ reported a higher level of knowledge on dental waste management among postgraduate students as compared to trainees and undergraduate students.

To date, only a handful of studies have investigated educational interventions on the management of biomedical waste and recycling and reuse in the dental profession^26,27^. Furthermore, the majority of these studies employed a cross-sectional design^6,11,24,25,28–30^. Therefore, the purpose of this study was to assess the effects of a virtual educational program on the knowledge and awareness of dental material recycling and reuse, as well as biomedical waste management, among dental professionals in Peru.

## Methods

### Study design

The study, conducted between April 2022 and January 2023, followed a longitudinal, analytical, prospective, and quasi-experimental design^31^. The participants were Peruvian dentists from both the capital city and a province in Peru.

### Population and selection of participants

The study population consisted of Peruvian dentists from either Lima or the province of Ica, both located on the Peruvian coast, who practiced in either the public or private healthcare sector, including some who also taught in universities. Additionally, the virtual educational program was administered during the final year of the Covid-19 pandemic when social distancing protocols were still mandatory in Peru. The study sample comprised 165 Peruvian dentists and was determined utilizing the EPIDAT 4.2 program and a formula for estimating unknown population proportions. Statistical data obtained from a prior pilot study with 50 participants provided the basis for the calculation, with *p* = 0.88 and q = 0.12, and a precision error of 5% and a significance level of α = 0.05. The sample selection method employed non-probabilistic intentional sampling, wherein a group of dentists meeting the eligibility criteria were invited to participate.

Dentists who provided informed consent, graduated from a Peruvian university, had virtual platform access, and engage in dental education and/or care were eligible. Excluded were those who withdrew consent, failed to complete the questionnaire at all three evaluation points, or had previous training in recycling and biomedical waste management.

### Sociodemographic variables

The present study considered age, sex, university of origin, marital status, place of origin, academic degree, specialization, work performed, and professional experience^24,25^.

### Validation of the instrument

A validated 30-item questionnaire^6^ with two dimensions was utilized, consisting of one section focused on managing biomedical waste (Q1–Q15), and another section on recycling and reusing dental materials (Q16–Q30). The questions were designed as closed-ended, offering three response options (Yes/No/Don't know) Three judges with more than 15 years of experience (two doctors in public health and a research professor in dentistry) validated the instrument's content for transculturality, pertinence, objectivity, relevance, timeliness, sufficiency, clarity, and methodology, achieving an acceptable Aiken's V score of 0.94 (CI 0.91–0.96). Correct answers received one point, while incorrect answers received zero points.

Cronbach's alpha was used to determine the internal consistency of the instrument, resulting in an (α) value of 0.80 (95% CI 0.75–0.84), indicating acceptable reliability. To assess the questionnaire's reproducibility, 30 randomly selected participants were surveyed at two different times within seven days and the question order was changed to avoid recall bias^32^ prior to the educational video being shown. The obtained scores had an acceptable intraclass correlation coefficient (ICC) of 0.96 (95% CI 0.91–0.98).

### Characteristics of the educational video

The educational intervention was conducted via a 9.27-min video hosted on YouTube (https://youtu.be/ScKKecVmcyg). The video provided vivid still and moving images in Spanish with a clear and audible delivery and a colorful interface against a white background marked with a light red line at the top. The presentation employed a laptop graphic and covered the management of biomedical waste, including hospital waste management in the dental field. A presentation was given on the recycling, reuse, and proper disposal of dental materials, including dental amalgam, mercury, gypsum, thermoplastic materials, elastomeric materials, plastics, silver, titanium, zirconium, nickel, and copper. Information was provided regarding proper waste management for dental X-rays, developer, and fixative liquids, which may contain hazardous chemical substances. Additionally, orientation was given on the various types of wastewater treatment processes. Law No. 1278 sets forth three fundamental pillars for the appropriate management of solid waste in Peru: prioritizing waste reduction, being efficient in the use of materials, and viewing waste as a resource rather than a threat. Additionally, the document provides general information on the reuse of needles and intravenous equipment, and highlights the emission of greenhouse gases resulting from improper waste incineration.

The video underwent validation by three expert judges consisting of a public health specialist, a research methodology faculty member, and an environmental engineer. They evaluated attractiveness, comprehension, compatibility, acceptability, relevance, and ability to induce action^33^. The result was an acceptable Aiken V value of 0.87 (95% CI: 0.83—0.90).

### Procedure

The questionnaire was created using the virtual Google Classroom® platform and shared with dentists via a web link on WhatsApp®. Clicking on the provided link directed participants to the informed consent form, containing the e-mail address, telephone number, and complete name of the principal investigator, as well as the university of origin and the ethics committee's institutional e-mail address. If participants provided their consent, the system directed them to the subsequent page where they received instructions to complete the questionnaire (pre-test). If participants failed to respond, the invitation was re-shared up to three times within 15 days. The researchers (CCR, NCL, MLC, GBV, JHE, PLC, and MCM) were responsible for sending the invitation. After obtaining consent to participate, the researcher contacted each participant to confirm that they had fully watched the video after completing the pre-test, whether through a Microsoft Teams meeting or in person via an electronic device. Afterward, the participant completed a repeated questionnaire (post-test). After a span of 14 days (± 1 day), the researcher shared the questionnaire with the participant once more through a weblink (post-test-2), either virtually or in person. Throughout the study, participants were given the option to decline the invitation, opt out of the questionnaire, or stop watching the video at any point. Data was accessed by CCR, GBV, MCM, PLC, and MLC, and encrypted for confidentiality on a password-protected digital device. Only one complete response was accepted per participant. To avoid duplicate responses, participants were required to indicate their first and last name along with their age (e.g., CCR41). This was to prevent overlapping replies in case someone accessed the questionnaire link from two different email addresses. Participants were not given any incentives to participate and were given access to the questionnaire link from April 10, 2022 to January 31, 2023.

### Data analysis

Data were recorded in a Microsoft Excel 2019 spreadsheet for later analysis using Statistical Package for the Social Sciences (SPSS) version 28.0. To conduct a descriptive analysis of the qualitative variables, both absolute and relative frequencies were used. In the case of the quantitative variable age, measures of central tendency such as mean and median were utilized along with measures of dispersion such as standard deviation and interquartile range. Ordinal variables were compared using the nonparametric Mann Whitney U test for two categories and the Kruskal Wallis H test for more than two categories at once. To compare related measures at three time points, the Friedman test with Bonferroni's post hoc test was used. The Cochrane's Q test with Bonferroni's post-hoc test was also used to compare the proportion of correct answers to the questionnaire questions at the three time points. Significance was considered at a level of p < 0.05 for all statistical tests.

### Ethical approval and consent to participate

The present study respected the bioethical principles of the Declaration of Helsinki related to freedom, nonmaleficence, respect and confidentiality^34^. In addition, this research had the approval of an Institutional Research Ethics Committee of the UPSJB with resolution No. 523–2022-CIEI-UPSJB dated April 8, 2022. In addition, on the first page of the virtual questionnaire, participants were asked to give voluntary informed consent.

## Results

The response rate of the participants was 59.35% and the average age was 32.9 ± 9.9 years. The percentage of those aged 30 years or younger was slightly higher than those older than 30 years, with a difference of less than 1%. The predominant sex was female with 57.0% of the total. 81.2% of the participants studied at a private university, 71.5% of the total were unmarried and 78.2% of the respondents were from the capital city. Also, 78.8% of the total had only a bachelor's degree and 81.2% did not have a specialization. Finally, 70.9% of the dentists were solely involved in private care with 81.2% of the total having less than 10 years of professional experience (Table 1).

**Table 1:** Sociodemographic characteristics of surveyed Peruvian dentists.

Variable	Category	Frequency	Percentage
Age group	≤ 30 years	83	50.3
	> 30 years	82	49.7
Sex	Female	94	57.0
	Male	71	43.0
University of origin	Public	31	18.8
	Private	134	81.2
Marital status	Unmarried	118	71.5
	Married or cohabiting	47	28.5
Place of origin	Capital	129	78.2
	Province	36	21.8
Academic degree	Bachelor	130	78.8
	Master	31	18.8
	Doctorate	4	2.4
Specialization	Yes	31	18.8
	No	134	81.2
Work performed	Public and private care	31	18.8
	Private care	117	70.9
	Teaching and care (public and/or private)	17	10.3
Professional Experience	< 10 years	134	81.2
	≥ 10 years	31	18.8
Age	Mean	Median	SD
	32.9	30.0	9.9

*SD* Standard deviation.

After comparing the proportion of accurate responses about biomedical waste management prior to and immediately following educational intervention via video, there was a noteworthy rise in knowledge across all inquiries (*p* < 0.05), except in Q3 (*p* = 0.063) and Q14 (*p* = 0.062), which had a frequency of correct answers higher than 90% at all times. In the remaining questions, there were no significant variations (*p* > 0.05) observed between the correct responses obtained 14 days following the educational intervention and those taken immediately after, except in Q11 (*p* = 0.010) (Table 2).

**Table 2:** Comparison of the proportion of correct answers about biomedical waste management over time.

Questionnaire	Correct answers			*p	Post hoc		
	Pre-test (X)	Post-test (Y)	14 days (Z)		(X) vs (Y)	(X) vs (Z)	(Y) vs (Z)
	f (%)	f (%)	f (%)		**p	**p	**p
Q1. Are you aware of government regulations and legislations related to biomedical waste management?	101 (61.2)	138 (83.6)	141 (85.5)	< 0.001	< 0.001	< 0.001	1.000
Q2. Are you aware of the theoretical and practical knowledge required to handle and/or recycle/reuse hospital waste?	119 (72.1)	160 (97.0)	154 (93.3)	< 0.001	< 0.001	< 0.001	0.965
Q3. Do you know that inadequate biomedical waste management contributes to environmental pollution and global warming?	156 (94.5)	162 (98.2)	162 (98.2)	0.063			
Q4. Do you know the six effective steps of biomedical waste management?	63 (38.2)	149 (90.3)	139 (84.9)	< 0.001	< 0.001	< 0.001	0.703
Q5. Do you remember the type of incinerator at the institution you studied at?	40 (24.2)	83 (50.3)	69 (41.8)	< 0.001	< 0.001	< 0.001	0.134
Q6. Do you know of other effective methods of waste disposal besides incineration and landfill?	77 (46.7)	122 (73.9)	124 (75.2)	< 0.001	< 0.001	< 0.001	1.000
Q7. In our country, is hospital waste being managed by trained professional personnel?	82 (49.7)	112 (67.9)	106 (64.2)	< 0.001	0.001	0.009	1.000
Q8. Do you know the wastewater treatment process?	82 (49.7)	137 (83.0)	128 (77.6)	< 0.001	< 0.001	< 0.001	0.767
Q9. Are you aware that lead vests and collars should be disposed of by authorized companies?	89 (53.9)	158 (95.8)	145 (87.9)	< 0.001	< 0.001	< 0.001	0.258
Q10. Did you know that faulty incineration emits greenhouse gases?	105 (63.6)	160 (97.0)	149 (90.3)	< 0.001	< 0.001	< 0.001	0.337
Q11. Do you know of any environmental technology that converts organic waste into commercially useful by-products?	36 (21.8)	124 (75.2)	99 (60.0)	< 0.001	< 0.001	< 0.001	0.010
Q12. Do you know the hazardous component of X-ray fixative solutions?	98 (59.4)	159 (96.4)	145 (87.9)	< 0.001	< 0.001	< 0.001	0.134
Q13. Do you feel that biomedical waste management should be a practice in dental school?	152 (92.1)	162 (98.2)	160 (97.0)	0.018	0.023	0.098	1.000
Q14. Are you aware that inappropriate management of biomedical waste affects the population?	150 (90.9)	158 (95.8)	158 (95.8)	0.062			
Q15. Do you feel that hospitals and other organizations are well equipped to handle biomedical waste?	31 (18.8)	58.0 (35.2)	65 (39.4)	< 0.001	< 0.001	< 0.001	0.957

*Based on Cochrane's Q test (*p* < 0.05, significant differences). **Post hoc with Bonferroni correction (*p* < 0.05, significant differences).

When comparing the percentage of correct responses about recycling and reuse of dental materials before and immediately after receiving educational intervention by video, a significant improvement in knowledge was found for all questionnaire items (*p* < 0.05), except for Q26 (*p* = 0.189), where the frequency of correct responses was consistently below 50%. In the other questions, no statistically significant differences (*p* > 0.05) were found between the correct answers at the immediate post-test and 14 days after receiving the educational intervention, except in Q20 (Table 3).

**Table 3:** Comparison of the proportion of correct answers about recycling and reuse of dental materials over time.

Questionnaire	Correct answers			*p	Post hoc		
	Pre-test (X)	Post-test (Y)	14 days (Z)		(X) vs (Y)	(X) vs (Z)	(Y) vs (Z)
	f (%)	f (%)	f (%)		**p	**p	**p
Q16. Are you aware that a component of dental amalgam constitutes an environmental risk?	152 (92.1)	162 (98.2)	161 (97.6)	0.008	0.015	0.034	1.000
Q17. Can silver be recovered from dental amalgam?	49 (29.7)	117 (70.9)	104 (63.0)	< 0.001	< 0.001	< 0.001	0.345
Q18. Can mercury be recovered from dental amalgam?	45 (27.4)	102 (61.8)	113 (68.5)	< 0.001	< 0.001	< 0.001	0.585
Q19. Have you seen a dental unit with an amalgam separator?	24 (14.5)	64 (38.8)	65 (39.4)	< 0.001	< 0.001	< 0.001	1.000
Q20. Should leftover amalgam used in a dental cavity be disposed of in a conventional cuspidor attached to the dental chair?	123 (74.5)	149 (90.3)	124 (75.2)	< 0.001	0.001	1.000	0.001
Q21. Are you aware that non-reusable materials such as syringes, needles and intravenous equipment can be recycled for other uses?	90 (54.5)	112 (67.9)	109 (66.1)	0.008	0.013	0.041	1.000
Q22. Can gypsum be recycled?	54 (32.7)	147 (89.1)	134 (81.2)	< 0.001	< 0.001	< 0.001	0.418
Q23. Can gypsum be used as land fill?	51 (30.9)	124 (75.2)	111 (67.3)	< 0.001	< 0.001	< 0.001	0.453
Q24. When used as land fill, can gypsum produce environmentally friendly gas?	36 (21.8)	98 (59.4)	99 (60.0)	< 0.001	< 0.001	< 0.001	1.000
Q25. Did you know that gypsum can be recycled for use in more than 10 different products?	39 (23.6)	124 (75.2)	120 (72.7)	< 0.001	< 0.001	< 0.001	1.000
Q26. Are you aware that elastomeric impression materials can be recycled?	65 (39.4)	79 (47.9)	78 (47.3)	0.189			
Q27. Are you aware that thermoplastic materials can be reused in dentistry?	71 (43.0)	149 (90.3)	132 (80.0)	< 0.001	< 0.001	< 0.001	0.126
Q28. Do you know what biodegradable plastic is?	129 (78.2)	154 (93.3)	149 (90.3)	< 0.001	< 0.001	0.002	1.000
Q29. Apart from dental gold, can other metals be reusable in dentistry?	102 (61.8)	147 (89.1)	134 (81.2)	< 0.001	< 0.001	< 0.001	0.263
Q30. Are you aware that more studies are needed related to the topic of recycling and reuse in dentistry?	153 (92.7)	164 (99.4)	160 (97.0)	< 0.001	0.001	0.052	0.523

*Based on Cochrane's Q test (*p* < 0.05, significant differences). **Post hoc with Bonferroni correction (*p* < 0.05, significant differences).

Significant differences were observed in the knowledge scores on biomedical waste management based on different sociodemographic variables before, immediately after, and 14 days after the educational intervention (*p* = 0.017, *p* = 0.002, and *p* = 0.015, respectively) between the dentists in the capital and the province. In addition, regarding academic degree, significant differences were found between bachelors and masters/doctors immediately after the educational intervention (*p* = 0.005). Also, a significant improvement in knowledge could be observed immediately after the educational intervention in all the sociodemographic variables considered in the study (*p* < 0.001). However, this improvement in knowledge about biomedical waste management was not significantly sustained 14 days after the educational intervention among those over 30 years of age (*p* = 0.042), who studied at a private university (*p* = 0.014), unmarried (*p* = 0.013), from the capital city (*p* = 0.002), with a bachelor's degree (*p* = 0.003), without a specialty (*p* = 0.004), working in public and private care (*p* = 0.047), and those with less than 10 years of professional experience (*p* = 0.024) (Table 4).

**Table 4:** Comparison of biomedical waste management knowledge scores between the categories of each variable and over time.

Variable	Category	Pre-test (X)			Post-test (Y)			14 days (Z)			**p	Post hoc		
												(X) vs (Y)	(X) vs (Z)	(Y) vs (Z)
		Median	IQR	*p	Median	IQR	*p	Median	IQR	*p		***p	***p	***p
Age group	≤ 30 years	9.0	3.0	0.684	13.0	2.0	0.242	12.0	3.0	0.063	< 0.001	< 0.001	< 0.001	0.143
	> 30 years	8.5	3.0		13.0	3.0		12.0	3.0		< 0.001	< 0.001	< 0.001	0.042
Sex	Female	9.0	3.0	0.939	13.0	3.0	0.508	12.0	2.0	0.508	< 0.001	< 0.001	< 0.001	0.071
	Male	8.0	3.0		13.0	3.0		12.0	3.0		< 0.001	< 0.001	< 0.001	0.087
University of origin	Public	10.0	2.0	0.099	12.0	2.0	0.992	12.0	2.0	0.520	< 0.001	< 0.001	< 0.001	0.547
	Private	8.0	3.0		13.0	3.0		12.0	2.0		< 0.001	< 0.001	< 0.001	0.014
Marital status	Unmarried	8.0	3.0	0.461	13.0	2.0	0.644	12.0	2.0	0.535	< 0.001	< 0.001	< 0.001	0.013
	Married or cohabiting	9.0	3.0		13.0	3.0		12.0	2.0		< 0.001	< 0.001	< 0.001	0.540
Place of origin	Capital	9.0	3.0	0.017	13.0	2.0	0.002	12.0	2.0	0.015	< 0.001	< 0.001	< 0.001	0.002
	Province	8.0	4.0		12.0	3.0		11.0	3.0		< 0.001	< 0.001	< 0.001	1.000
Academic degree	Bachelor	9.0	3.0	0.720	13.0	2.0	0.005	12.0	2.0	0.344	< 0.001	< 0.001	< 0.001	0.003
	Master/Doctor	9.0	3.0		12.0	2.0		12.0	3.0		< 0.001	< 0.001	< 0.001	1.000
Specialization	Yes	9.0	2.0	0.149	13.0	3.0	0.760	13.0	3.0	0.089	< 0.001	< 0.001	< 0.001	1.000
	No	8.0	3.0		13.0	2.0		12.0	2.0		< 0.001	< 0.001	< 0.001	0.004
Work performed	Public and private care	9.0	2.0	0.154	13.0	2.0	0.060	13.0	2.0	0.346	< 0.001	< 0.001	< 0.001	0.047
	Private care	8.0	4.0		13.0	3.0		12.0	2.0		< 0.001	< 0.001	< 0.001	0.079
	Teaching and care (public and/or private)	10.0	3.0		12.0	2.0		12.0	2.0		< 0.001	< 0.001	< 0.001	1.000
Professional experience	< 10 years	8.5	3.0	0.215	13.0	2.0	0.300	12.0	2.0	0.201	< 0.001	< 0.001	0.002	0.024
	≥ 10 years	9.0	4.0		13.0	3.0		12.0	3.0		< 0.001	< 0.001	< 0.001	0.259

*Based on the Mann Whitney U to compare two categories and on the Kruskal Wallis H to compare three categories (*p* < 0.05, significant differences). **Based on Friedman's test (*p* < 0.05, significant differences); ***Based on post hoc with Bonferroni correction (*p* < 0.05, significant differences).

Significant differences were observed when comparing the scores of knowledge about recycling and reuse of dental materials before the educational intervention and according to the categories of each sociodemographic variable. These differences were found between individuals aged 30 years or less and those older than 30 years (*p* = 0.033), unmarried and married individuals (*p* = 0.021), specialists and non-specialists (*p* = 0.005), and between those who only worked in private care and those who worked in teaching and public/private care (*p* = 0.047). Immediately after the educational intervention, significant differences were observed between dentists from the capital and from the province (*p* < 0.001), between bachelors and professors/doctors (*p* = 0.002), between those who worked only in care and those who worked in care and teaching (*p* = 0.045), and between those with less than 10 years of experience and those with 10 years or more (*p* = 0.038).

After 14 days of receiving the educational intervention, there were significant differences between the dentists aged 30 years or less and those over 30 years (*p* = 0.009), and between those from the capital and those from the province (*p* = 0.003). On the other hand, a significant improvement in knowledge could be observed immediately after the educational intervention in all the sociodemographic variables considered in the study (*p* < 0.05). However, this improvement in knowledge about recycling and reuse of dental materials was not significantly sustained 14 days after the educational intervention for dentists aged 30 years or younger (*p* = 0.028), older than 30 years (*p* = 0.008), men (*p* = 0. 002), who studied at a private university (*p* < 0.001), unmarried (*p* = 0.001), with a bachelor's degree (*p* < 0.001), without specialty (*p* = 0.001), those who worked in the profession without teaching (*p* < 0.05) and those who had less than 10 years of experience (*p* < 0.001) (Table 5).

**Table 5:** Comparison of knowledge scores about recycling and reuse of dental materials between the categories of each variable and over time.

Variable	Category	Pre-test (X)			Post-test (Y)			14 days (Z)			**p	Post hoc		
												(X) vs (Y)	(X) vs (Z)	(Y) vs (Z)
		Median	RIQ	*p	Median	RIQ	*p	Median	RIQ	*p		***p	***p	***p
Age group	≤ 30 years	7.0	3.0	0.033	12.0	2.0	0.128	12.0	2.0	0.009	< 0.001	< 0.001	< 0.001	0.028
	> 30 years	7.5	5.0		12.0	3.0		11.0	3.0		< 0.001	< 0.001	< 0.001	0.008
Sex	Female	7.0	4.0	0.878	12.0	2.0	0.281	11.0	2.0	0.882	< 0.001	< 0.001	< 0.001	0.059
	Male	7.0	4.0		12.0	2.0		11.0	2.0		< 0.001	< 0.001	< 0.001	0.002
University of origin	Public	7.0	3.0	0.428	12.0	2.0	0.438	11.0	3.0	0.458	< 0.001	< 0.001	< 0.001	1.000
	Private	7.0	4.0		12.0	2.0		11.0	2.0		< 0.001	< 0.001	< 0.001	< 0.001
Marital Status	Unmarried	7.0	4.0	0.021	12.0	2.0	0.159	11.0	2.0	0.766	< 0.001	< 0.001	< 0.001	0.001
	Married or cohabiting	8.0	4.0		12.0	3.0		11.0	2.0		< 0.001	< 0.001	< 0.001	0.190
Place of origin	Capital	7.0	5.0	0.324	12.0	2.0	< 0.001	11.0	2.0	0.003	< 0.001	< 0.001	< 0.001	< 0.001
	Province	7.0	4.0		11.0	4.0		10.0	4.0		< 0.001	< 0.001	< 0.001	1.000
Academic degree	Bachelor	7.0	4.0	0.195	12.0	2.0	0.002	11.0	2.0	0.525	< 0.001	< 0.001	< 0.001	< 0.001
	Master/Doctor	7.0	3.0		11.0	4.0		11.0	3.0		< 0.001	< 0.001	< 0.001	1.000
Specialization	Yes	9.0	4.0	0.005	12.0	2.0	0.328	11.0	3.0	0.303	< 0.001	< 0.001	< 0.001	0.432
	No	7.0	3.0		12.0	2.0		11.0	2.0		< 0.001	< 0.001	< 0.001	0.001
Work performed	Public and private care	7.0 ^A,B^	3.0	0.040	12.0 ^A^	2.0	0.038	11.0	3.0	0.131	< 0.001	< 0.001	0.001	0.028
	Private care	7.0 ^A^	4.0		12.0 ^A,B^	2.0		11.0	2.0		< 0.001	< 0.001	< 0.001	0.006
	Teaching and care (public and/or private)	8.0 ^B^	5.0		11.0 ^B^	3.0		11.0	3.0		0.006	0.014	0.119	1.000
Professional experience	< 10 years	7.0	4.0	0.141	12.0	2.0	0.038	11.0	2.0	0.581	< 0.001	< 0.001	< 0.001	< 0.001
	≥ 10 years	7.0	3.0		12.0	3.0		11.0	3.0		< 0.001	< 0.001	0.001	1.000

*Based on the Mann Whitney U to compare two categories and on the Kruskal Wallis H to compare three categories (*p* < 0.05, significant differences). **Based on Friedman's test (*p* < 0.05, significant differences); ***Based on post hoc with Bonferroni correction (*p* < 0.05, significant differences).

## Discussion

According to the WHO, 15% of waste produced by health facilities is classified as hazardous and can be infectious, toxic, or radioactive^30^. Dental procedures generate a significant amount of biomedical waste, which can negatively impact the environment and contribute to climate changes^3,29^. Consequently, it is vital for dentists to be cognizant of safe waste disposal practices and understand recycling and reusing dental materials to mitigate harmful effects. the purpose of this study was to assess the effects of a virtual educational program on the knowledge and awareness of dental material recycling and reuse, as well as biomedical waste management, among dental professionals in Peru.

The present study’s findings show that after receiving educational intervention through a video, there was a significant increase in knowledge about biomedical waste management when comparing the proportion of correct answers before and immediately after the intervention. These findings align with the significant increase in healthcare professionals' knowledge observed in studies by Kumar et al.^35^, Kaore et al.^36^, and Conde et al.^37^ following the implementation of educational interventions on biomedical waste management. This study showcases the significance of educational intervention in increasing the awareness of healthcare professionals and improving their understanding of the management of waste generated during the healthcare process^37,38^. Questionnaire results revealed over 90% accuracy in responses to Q3 and Q14, indicating that dentists were consistently cognizant of the adverse impacts of inadequate management of biomedical waste on the environment, population, and global warming. The viewpoints of Kaore et al.^36^ and Conde et al.^37^ corroborate the latter's argument. Similarly, our study aligns with Karki et al.'s research^39^, which revealed that roughly 90% of health care workers surveyed at both public and private hospitals in Nepal understood that improper management of health care waste could contaminate water and air and thereby harm the environment. It is noteworthy that this was a cross-sectional study that did not entail any interventions. These findings may be attributed to the necessity for healthcare professionals to obtain specific job-related skills and competencies in order to practice their profession and secure an operating license or municipal permit in either the public or private sectors. Consequently, they engage in self-directed training. However, the findings differ from those reported by Tilahun et al.^40^, who discovered that 61.7% of healthcare staff in the Oromia region (Ethiopia) were cognizant that the inappropriate disposal of medical waste contributes to the spreading of diseases. This difference may be attributed to the study by Tilahun et al.^40^ being conducted in private healthcare facilities where practitioners frequently seek personal guidance and support until gaining legal authorization to practice. As a result, practitioners may believe that they are adequately delivering high-quality medical care with all necessary biosafety measures in place prior to and during patient care, thereby downplaying the significance of appropriate disposal of biomedical waste.

On the other hand, when comparing the correct answers regarding biomedical waste management 14 days after the intervention, there were no significant differences with the immediate posttest, except for Q11. This indicates that knowledge about the environmental technology that transforms organic waste into commercially valuable by-products was not significantly maintained 14 days after receiving the educational intervention. This may be due to the novelty of the subject for dentists, as it is not yet included in the curricula of most Peruvian dental schools, and therefore they do not receive any formal training on the subject during their academic studies. In addition, the dental profession prioritizes the clinical aspect to train manual dexterity, often at the expense of appropriately managing the biomedical waste produced by dental procedures and raising awareness of its negative impact on the environment^3,6,11,41–44^. After comparing the rates of accurate responses regarding dental material recycling and reuse before and immediately after educational intervention, a considerable improvement in knowledge was observed across all questionnaire items, except for Q26, where correct responses consistently fell below 50%. These results suggest that the educational video alone did not significantly enhance dentists' understanding of recycling elastomeric materials utilized in dental impressions. This may be attributed to the video being shown only once, which is insufficient to sustain knowledge over time. This finding supports Oke and Kruijsen's study^45^, which found that knowledge returns to its initial state shortly after the removal of a short-term intervention. Additionally, it is noteworthy that dental technicians are typically responsible for removing or reusing elastomeric materials, as they receive the work from dental professionals in order to complete the corresponding laboratory process^25,46^. This situation may have caused dental technicians to disregard or minimize the significance of this section in the educational video.

Upon comparing the accurate responses regarding the recycling and reuse of dental materials 14 days following the intervention, it was noted that there were no noteworthy distinctions from the immediate post-test, apart from Q20. This implies that knowledge about proper disposal of surplus amalgam was not retained significantly for 14 days post receiving the educational intervention. The decline in the use of amalgam in dental restorations as a result of the Minamata Convention on Mercury^47–49^ may have led to a downplaying of the importance of this issue. In addition, studies reporting possible adverse effects on the environment and human health have led to the omission of this topic from professional training courses^50,51^. However, it is still possible to extract and discard amalgams from patients who frequently visit the dental office in Peru with this type of restoration. This can occur either as a replacement with resin composite or due to partial fracture of the cavity walls.

Based on the current study's findings, dentists in the capital city exhibit greater knowledge and awareness of recycling, reusing dental materials, and managing biomedical waste compared to their provincial counterparts. This may be attributed to the extensive regulation of private professional practice in the Peruvian capital by government authorities, which has influenced the handling of biomedical waste and its environmental impact. Consequently, dentists may prioritize acquiring knowledge on biomedical waste management to avoid any fines or suspensions of their operating license. This situation differs considerably in province regions because governmental oversight and enforcement of environmental laws are less strict or have a certain degree of leniency, likely due to the informality of marginal urban areas and the requirement for healthcare staff in areas far from the capital^52,53^.

Biomedical waste management is critical in dental practices because health care workers are constantly exposed to various physical, chemical, biological, and environmental hazards that can result in bacterial and parasitic infections and sharp injuries^54,55^. Therefore, proper management of biomedical waste is essential to reduce these risks^54,55^. Even polychlorinated biphenyls and dioxins, which are known to cause cancer, are released into the environment during incineration^56^. Moreover, the outdoor storage of biomedical waste poses a high risk of environmental contamination because it can release a large amount of harmful gases such as methane and sulfur, as well as radioactive materials, which significantly pollute the atmosphere^56^. Pathogens, heavy metals, and organic pollutants found in dental waste may cause surface and groundwater pollution, leading to changes in soil properties and accumulation of heavy metals that can harm the ecosystem^56^. As dental professionals aim to prevent and treat oral health conditions to promote human health, it is crucial to preserve the ecological balance to ensure the long-term sustainability of the planet. Therefore, effective planning, knowledge, practice, and adaptation of waste management procedures in medical activities are crucial to mitigate the aforementioned inherent risks^57^.

One limitation of the study is that knowledge was only evaluated up to 14 days after the intervention. To better assess the impact of educational interventions, it is recommended that knowledge be evaluated up to 6 months or 1 year post-intervention. Previous research has shown that such interventions can sensitize health professionals, resulting in positive changes to their attitudes and practices^37^. Another possible limitation to consider is the utilization of purposive sampling, which does not permit us to draw significant generalizations on the population level. Nonetheless, it establishes a foundation for developing forthcoming research aimed at promoting environmental consciousness among students and health practitioners. This sampling limitation resulted from the present longitudinal study's recruitment of dentists who volunteered to participate and complete the questionnaire during the three points assessed. Furthermore, despite intentional invitations being made in various parts of the capital and a Peruvian province by seven researchers, some participants did not complete the third evaluation within the proposed timeframe. As a result, their examinations needed to be discarded, and the researchers had to wait ten months to recruit additional participants to achieve the minimum statistically required sample size. This was shown by a response rate of 59.35% among all dentists invited to take part in the study.

Based on the obtained results, it is essential to recommend that dental schools integrate into their curricula courses that instruct students on social responsibility and environmental care issues^58^. This should be included in the curriculum from the first year of dental studies to raise awareness among future dentists about recent advancements in waste disposal. Proper waste management is crucial for minimizing harmful environmental effects, and every inhabitant of the planet bears this responsibility. Structured videos with clear beginnings, developments, and endings, featuring a summary of the main ideas, can be successfully leveraged as educational strategies for courses involving biomedical waste. Furthermore, it is recommended that dentists be made aware of novel techniques for recycling and reusing waste via their dental professional associations^25,59^. Finally, it is recommended that government entities encourage research through educational interventions to assess the sustainability and environmental impact of dental practices. This will allow oral health professionals to comprehend, oversee, and evaluate their impacts. Additionally, future studies should include topics that involve the safety measures utilized by healthcare workers against biomedical waste.

## Conclusion

Dentists demonstrated a significant improvement in their understanding and awareness of biomedical waste disposal and dental materials recycling and reuse immediately following the educational intervention, and this was observed across all sociodemographic categories studied in this study. However, this knowledge this information was not retained after a period of two weeks among individuals who studied at a private university, unmarried, bachelor, with no specialty, non-teachers and those with less than 10 years of professional experience.

## Data Availability

All data analyzed during this study are available from the corresponding author on reasonable request.

## Abbreviations

CI: Confidence interval
HBV: Hepatitis B
HCV: Hepatitis C
HIV: Human immunodeficiency virus
SARS-CoV-2: Severe acute respiratory syndrome coronavirus 2
SPSS: Statistical package for the social sciences
WHO: World Health Organization
